# Clinically recognized sepsis in older adults admitted to medical wards: patient characteristics and hospital outcomes from the REPOSI register

**DOI:** 10.1007/s11739-026-04501-z

**Published:** 2026-08-24

**Authors:** Marta Chiara Sircana, Alfredo Caturano, Mauro Tettamanti, Sara Ratti, Chiara Ronchetti, Alessandro Nobili, Francesca Innocenti, Pier Mannuccio Mannucci, Roberto Manetti, REPOSI Investigators, Marta Chiara Sircana, Mauro Tettamanti, Sara Ratti, Alessandro Nobili, Pier Mannuccio Mannucci, Roberto Manetti, Nicola Montano, Giorgio Sesti, Antonello Pietrangelo, Antonio De Vincentis, Alessandra Marengoni, Luca Pasina, Carlotta Franchi, Alessia Antonella Galbussera, Sara Mandelli, Ilaria Ardoino, Alessio Novella, Enrico Nicolis, Domenico Prisco, Elena Silvestri, Giacomo Emmi, Irene Mattioli, Matteo Mazzetti, Edoardo Biancalana, Gianni Biolo, Giacomo Bartelloni, Ilaria Martini, Matteo Pirro, Graziana Lupattelli, Vanessa Bianconi, Riccardo Alcidi, Alessia Giotta, Massimo R. Mannarino, Domenico Girelli, Fabiana Busti, Giacomo Marchi, Nicola Veronese, Mario Barbagallo, Ligia Dominguez, Vincenza Beneduce, Federica Cacioppo, Stefano Ciriminna, Andrea Asaro, Chiara Giannettino, Claudia Canizzo, Giovanna Ottavia Plano, Anna Fazzari, Alessandra Lo Nigro, Ilenia Imbraguglio, Sofia Contorno, Salvatore Corrao, Giuseppe Natoli, Salvatore Mularo, Valentina Orlando, Giacomo Corrao, Fabio Falcone, Salvatore Scibetta, Luigi Mirarchi, Simona Amodeo, Brenda Bongiorno, Marco Zoli, Maria Laura Matacena, Giuseppe Orio, Eleonora Magnolfi, Giovanni Serafini, Mattia Brunori, Ilaria Lazzari, Angelo Simili, Tiziano Lucchi, Paola Nicolini, Gabriele Ghidini, Miriana Martelengo, Maddalena Fabrizi, Giulia Vigani, Arturo Cerizza, Rita Deda, Miriam Zappa, Antonio Di Sabatino, Emanuela Miceli, Marco Vincenzo Lenti, Lavinia Pitotti, Valentina Antoci, Ginevra Cambiè, Costanza Caccia Dominioni, Roberto Pontremoli, Valentina Beccati, Giulia Nobili, Giovanna Leoncini, Jacopo Alberto, Federico Cattaneo, Francesco Cipollone, Maria Teresa Guagnano, Ilaria Rossi, Damiano D’Ardes, Alessia Cipollone, Riccardo Mattia Ricciardi, Marco Bucci, Paola Vizzarri, Gerardo Mancuso, Daniela Calipari, Mosè Bartone, Maria Berria, Alessandro Delitala, Pierluigi Meloni, Silvia Masala, Maurizio Muscaritoli, Alessio Molfino, Antonella Giorgi, Christian Gracin, Giovanni Imbimbo, Carmen Gallicchio, Ottavio Martellucci, Antonietta Gigante, Chiara D’Elia, Thelma Geneletti, Eleonora Carlotti, Arianna Pagani, Claudia Capelli, Antonio Picardi, Umberto Vespasiani Gentilucci, Paolo Gallo, Jessica Avagliano, Francesca Terracciani, Giuseppe Bellelli, Maurizio Corsi, Lavinia Vitali, Armida Soccali, Franco Arturi, Elena Succurro, Melania Melina, Federica Giofrè, Francesca Cosentino, Delia Chiarello, Francesco Andreozzi, Antonio Brucato, Teresa De Falco, Enrica Negro, Martino Brenna, Lucia Trotta, Giovanni Lorenzo Squintani, Giacomo Iacomelli, Giulia Colazzo, Paolo Simioni, Irene Bertozzi, Sara Angela Malerba, Camilla Portinari, Giordano Antonello, Cecilia Fortino, Camilla Goffi, Francesca Guidolin, Roberto Manfredini, Benedetta Boari, Alfredo De Giorgi, Ruana Tiseo, Giulia Marta Viglione, Caterina Savriè, Giuseppe Paolisso, Maria Rosaria Rizzo, Claudia Catalano, Irene Di Meo, Michele Cerasuolo, Claudio Borghi, Federica Piani, Federico Ruscelli, Chiara Baldini, Giulia Boni, Carlo Sabbà, Francesco Saverio Vella, Patrizia Suppressa, Alessio Comitangelo, Emanuele Amoruso, Carlo Custodero, Giuseppe Re, Chiara Maria Palmisano, Luigi Fenoglio, Giulia Racca, Alessia Valentina Giraudo, Salvatore D’Agnano, Giorgia Sasia, Irene Ruocco, Floriana Mao, Alice Ferrua, Anna Ludovica Fracanzani, Silvia Tiraboschi, Annalisa Cespiati, Giovanna Oberti, Giordano Sigon, Felice Cinque, Lucia Colavolpe, Jaqueline Currà, Francesca Alletto, Natalia Scaramellini, Simona Leoni, Alessandra Danuta Di Mauro, Gianpaolo Benzoni, Margherita Re, Laura Vivi, Valentina Scime, Flora Peyvandi, Raffaella Rossio, Giulia Colombo, Pasquale Agosti, Erica Pagliaro, Ciro Canetta, Valeria Savojardo, Giuliana Ceriani, Christian Folli, Fabrizio Montecucco, Cristina Michelauz, Elisa Schiavetta, Chiara Olivero, Simone Isoppo, Curzia Tortorella, Federico Carbone, Luca Liberale, Aurora Mazzocato, Nicola Lucio Liberato, Tiziana Tognin, Francesco Purrello, Antonino Di Pino, Salvatore Piro, Maurizio Di Marco, Nicoletta Miano, Sabrina Scilletta, Renzo Rozzini, Francesca Mazzeo, Giulia Cono, Giulia Cesaroni, Giuseppe Montrucchio, Paolo Peasso, Edoardo Favale, Cesare Poletto, Carl Margaria, Maura Sanino, Valeria Bianchi, Beatrice Bovo, Beatrice Brusasco, Gabriele Giuliano, Marco Angeleri, Luigina Guasti, Francesca Rotunno, Luana Castiglioni, Andrea Maresca, Leonardo Campiotti, Alessandra Grossi, Roberto Davide Diprizio, Francesco Dentali, Veronica Behnke, Gloria Cuni, Marco Bertolotti, Chiara Mussi, Giulia Lancellotti, Laura Orlandi, Federica Di Zio, Gabriele Luppi, Francesca D’Imprima, Chiara Mazza, Claudia Grandi, Chiara Ferrari, Ambrogio Breschi, Angela Sciacqua, Maria Perticone, Raffaele Maio, Aleandra Scozzafava, Valentino Condoleo, Elvira Clausi, Giuseppe Armentaro, Alberto Panza, Alessia Grandinetti, Carlo Alberto Pastura, Gianluca Moroncini, Emanuele Filippini, Devis Benfaremo, Pietro Castellino, Luca Zanoli, Agostino Gaudio, Anastasia Xourafa, Concetta Spichetti, Serena Torre, Alfio Gennaro, Lucrezia Taibi, Francesca Ferrari, Guido Moreo, Silvia Prolo, Gloria Pina, Lorenzo Magni, Sophia Ratzinger, Alberto Ballestrero, Fabio Ferrando, Roberta Gonella, Arianna Piccardo, Andrea Bellodi, Dejvis Kalaja, Anna Linda Patti, Giuseppe Famularo, Patrizia Tarsitani, Tiziana Morretti, Andrea Aglitti, Marcello Giuseppe Maggio, Fulvio Lauretani, Marco Salvi, Chiara Cattabiani, Elena Bronzoni, Aida Hoxha, Beatrice Tanzi, Stefano Del Giacco, Davide Firinu, Giulia Costanzo, Andrea Giovanni Ledda, Matteo Benuzzi, Salvatore Chessa, Anna Licata, Giuseppe Montalto, Filippo Alessandro Montalto, Angelo Rizzo, Silvia Como, Valentina Virzì, Giorgio Basile, Antonino Catalano, Federica Bellone, Concetto Principato, Angelo Cocuzza, Annamaria Buda, Francesco Corica, Lorenzo Malatino, Benedetta Stancanelli, Valentina Terranova, Salvatore Di Marca, Rosario Di Quattro, Lara La Malfa, Rossella Caruso, Ivan Isaia, Federica Castelletti, Matteo Regolo, Clara Salmeri, Nicolas Cardaci, Alessandra Lanzafame, Patrizia Mecocci, Carmelinda Ruggiero, Virginia Boccardi, Pietro Minuz, Luigi Fondrieschi, Sarah Morellini, Andrea Sartorio, Mario Pirisi, Gian Paolo Fra, Daniele Sola, Mattia Bellan, Emilio Simeone, Rosa Scurti, Fabio Tolloso, Roberto Tarquini, Alice Valoriani, Silvia Dolenti, Giulia Vannini, Francesco Cei, Marco Gucci, Antonella Fardus Al Refaie, Virginia Morelli, Fabio Salvadori, Sergio Harari, Chiara Lonati, Federico Napoli, Italia Aiello, Gabriele Tarallo, Chiara Apostolico, Ferdinando Carlo Sasso, Teresa Salvatore, Lucio Monaco, Carmen Ricozzi, Francesca Coviello, Maria Elena Corona, Giovanni Di Lorenzo, Serafina Schettino, Christian Catalini, Davide Nilo, Ilaria Indiano, Alberto Pilotto, Federica Gandolfo, Davide Gonella, Moreno Tresoldi, Enrica Bozzolo, Sarah Damanti, Gaia Deonette, Giulia Marazzi, Rita Venezia, Lorenzo Ciocca, Jasmin Mahajne, Chiara Calabrese, Giusy Faggiano, Marta Secci, Martina Laffranchi, Ilaria Vergine, Alessandro Marinosci, Cecilia Bussolari, Giovanni Benanti, Francesca Conti, Filippo Sciammetta, Rabia Saeedbhatti, Paolo Pasquero, Massimo Porta, Miriam Gino, Stefania Morra Di Cella, Bianca Pari, Edoardo Pace, Alessandro De Salve, Patrizia Tilocca, Sebastiana Maria Atzori, Alberto Moggi Pignone, Giulia Bandini, Maria Cristina De Santis, Chiara Di Cencio, Anna Lo Cricchio, Maria Lorenza Muiesan, Carolina De Ciuceis, Stefano Tenore, Fabio Cherubini, Francesco Russomanno, Nicola Liuzzi, Sokol Rrodhe, Alice Parisi, Eleonora Croce, Fiammetta Pagnozzi, Emanuela Messa, Francesca Gaia Bacchieri, Gaetano Serviddio, Antonino Davide Romano, Gianluigi Vendemiale, Francesco Bellanti, Aurelio Lo Buglio, Antonino Tuttolomondo, Domenico Di Raimondo, Edoardo Pirera, Riccardo De Rosa, Francesca Dassie, Angelo Di Vincenzo, Sara Brandolese, Agostino Di Ciaula, Antonino Noto, Chiara Valentina Luglio, Piero Portincasa, Leonardo A. Sechi, Cristiana Catena, Gabriele Brosolo, Stefano Marcante, Andrea Da Porto, Luca Bulfone, Antonio Vacca, Angelo Baldassare Cefalù, Antonina Giammanco, Carola Maria Gagliardo, Elena Campello, Chiara Simion, Michela Zanetti, Paolo De Colle

**Affiliations:** 1 Department of Medicine, Surgery and Pharmacology, University of Sassari, 07100 Sassari, Italy; 2 Department of Human Sciences and Promotion of the Quality of Life, San Raffaele Roma University, 00166 Rome, Italy; 3 Department of Health Policy, Istituto di Ricerche Farmacologiche Mario Negri IRCCS, Via Mario Negri 2, 20156 Milan, Italy; 4 Center for Advanced Simulation in Medicine, University Hospital Careggi, Lg. Brambilla 3, 50134 Florence, Italy; 5 A. Bianchi Bonomi Hemophilia and Thrombosis Center, IRCCS Ca’ Granda Maggiore Hospital Foundation, and University of Milan, Milan, Italy; 6 Present Address: Department of Clinical Research, University of Bern, Bern, Switzerland

**Keywords:** Sepsis, Older adults, Internal medicine, Multimorbidity, Frailty, Latent class analysis

## Abstract

Older adults admitted to internal medicine wards represent a growing population at high risk of sepsis, characterized by frailty, multimorbidity, functional impairment and reduced physiological reserve. While sepsis outcomes have been extensively studied in intensive care settings, less is known about prognostic determinants in non-ICU wards. Using data from the Italian REPOSI registry (2009–2023), we analyzed patients aged ≥65 years hospitalized in internal medicine and geriatric wards. Clinically recognized sepsis was identified through documented clinical diagnoses and corresponding ICD-9 codes. Latent Class Analysis (LCA) was used to explore multimorbidity patterns within the sepsis cohort. The primary aim was to evaluate factors associated with in-hospital mortality in older adults with sepsis. Secondary analyses compared baseline characteristics between septic and non-septic cases, assessed in-hospital mortality determinants according to sepsis onset setting (community- vs hospital-acquired), and explored factors associated with prolonged length of stay (LOS) in community-acquired sepsis. Additional descriptive analyses evaluated geographic distribution, infection sources and treatment patterns. Among 8880 patients, 504 (5.7%) had clinically recognized sepsis; more than half were hospital-acquired. In-hospital mortality was high (23.8%) and markedly higher in hospital-acquired sepsis. Two distinct multimorbidity patterns emerged: an oncologic–hepatologic profile and a diffuse chronic multimorbidity profile characterized by cardio–renal–metabolic, respiratory, and neuropsychiatric conditions. In community-acquired sepsis, the chronic multimorbidity profile was independently associated with increased mortality, whereas this association was not observed in hospital-acquired sepsis. Across the cohort, advanced age, functional dependence and hypoalbuminemia emerged as key factors independently associated with in-hospital mortality. Exploratory LOS analyses in community-acquired sepsis showed a modest association between prolonged hospitalization and higher anticholinergic burden. Additional descriptive analyses showed that lower respiratory tract infections were the most frequent source of both community- and hospital-acquired sepsis, while hospital-acquired cases showed greater heterogeneity of infectious sources and treatment patterns. In internal medicine wards, clinically recognized sepsis in older adults may reflect underlying clinical vulnerability rather than an isolated acute event. Multimorbidity patterns and geriatric-related clinical dimensions appear to influence outcomes, particularly in community-acquired sepsis. Integrating multimorbidity, nutritional and functional assessments into sepsis evaluation may improve risk stratification and support more personalized management in older hospitalized patients.

**Supplementary Information:**

The online version contains supplementary material available at https://doi.org/10.1007/s11739-026-04501-z.

## Introduction

Sepsis is a major clinical challenge in internal medicine wards, where the hospital population is predominantly older, multimorbid and clinically vulnerable [1, 2]. In this setting, sepsis often develops subacutely, with subtle and atypical features that may delay diagnosis and treatment in patients with a complex clinical pattern, where organ dysfunction intertwines with frailty, comorbidities, functional impairment and iatrogenic factors [3].

Although extensively investigated in intensive care units (ICUs), the burden of sepsis in internal medicine wards remains relatively underexplored, despite high mortality, disability, and prolonged hospital stays [4, 5].

Emerging evidence indicates that sepsis is a biologically heterogeneous condition with distinct clinical phenotypes influenced by the type of pathogens, infection sources and comorbidity patterns. This heterogeneity contributes to variable outcomes, underscoring the need for integrated stratification models capturing clinical complexity, multimorbidity and geriatric vulnerability.

At an epidemiological level, both incidence and mortality of sepsis are increasing in Europe, with an estimated 400,000 − 800,000 sepsis cases each year and a fatality rate between 15 and 25% [5]. In Italy, sepsis-related deaths increased from 18,939 (3%) in 2003 to 49,010 (8%) in 2015, in parallel with population aging, multimorbidity, invasive procedures, and antimicrobial resistance [6].

The Sepsis-3 definition framed sepsis as a life-threatening organ dysfunction caused by a dysregulated host response to infection [7]. However, in older adults, commonly used scores (qSOFA, SOFA, SIRS, NEWS) rely heavily on vital signs and show limited sensitivity in this age group, where physiological responses are often blunted, and they fail to capture functional reserve [8, 9].

Geriatric domains including multimorbidity, nutritional and functional status are strong prognostic determinants and should be incorporated into risk stratification [10–12]. Since vulnerability is driven by specific combinations of comorbidities rather than their mere number, person-centered analytical approaches such as the Latent Class Analysis (LCA) are particularly suitable [13].

From these premises and gaps of knowledge, the present study, based on the Italian REPOSI registry, aimed to characterize clinically recognized sepsis in older adults hospitalized in internal medicine wards, with particular focus on multimorbidity patterns and in-hospital mortality. Secondary analyses explored determinants of in-hospital mortality according to sepsis onset (community-acquired vs hospital-acquired) and factors associated with prolonged length of stay in community-acquired sepsis, while additional descriptive analyses assessed infection sources, antimicrobial treatment patterns, and geographical distribution within the registry.

## Methods

### Study design

This retrospective study is based on data from the REPOSI (REgistro POliterapie SIMI) registry, an ongoing collaborative initiative launched in 2008 by the Italian Society of Internal Medicine (SIMI), the Istituto di Ricerche Farmacologiche Mario Negri IRCCS, and the Fondazione Ospedale Policlinico IRCCS Ca’ Granda Maggiore. The registry involves multiple internal medicine and geriatric hospital wards across Italian regions. Detailed information on the REPOSI project has been reported in Mannucci et al. [14].

Patients aged 65 years and older were recruited. Recruitment took place during four predefined index weeks each year. Written informed consent was obtained from all participants prior to enrolment. The study protocol was approved by the ethics committee of each participating center. Information on health status, clinical events and drug therapy were recorded at admission, during hospital stay, and at discharge. Functional status (Barthel Index), nutritional status as estimated by serum albumin, and other clinical and laboratory variables were assessed at hospital admission and used as baseline indicators for the analyses. Frailty was assessed using a Frailty Index based on the deficit accumulation approach proposed by Rockwood and Mitnitski and previously implemented in the REPOSI registry [15, 16]. The index incorporates socio-demographic, physical, cognitive, functional, and clinical deficits and provides a quantitative measure of biological vulnerability, with higher scores indicating greater frailty. The REPOSI registry collects clinical and laboratory data at admission and at discharge from internal medicine wards and does not capture clinical status at sepsis onset. Consequently, available data do not allow assessment of acute illness severity at sepsis onset (e.g., full SOFA score), because in patients with sepsis present at admission they reflect post–emergency department conditions, no measurements being available at the onset of hospital-acquired sepsis. All diseases were classified according to the International Classification of Diseases, 9th revision (ICD-9) and all drugs were coded using the Anatomical Therapeutic Chemical (ATC) classification system [17, 18].

The initial analytical cohort comprised 10,253 individuals enrolled in the REPOSI registry between 2008 and 2023.

**Fig. 1:**
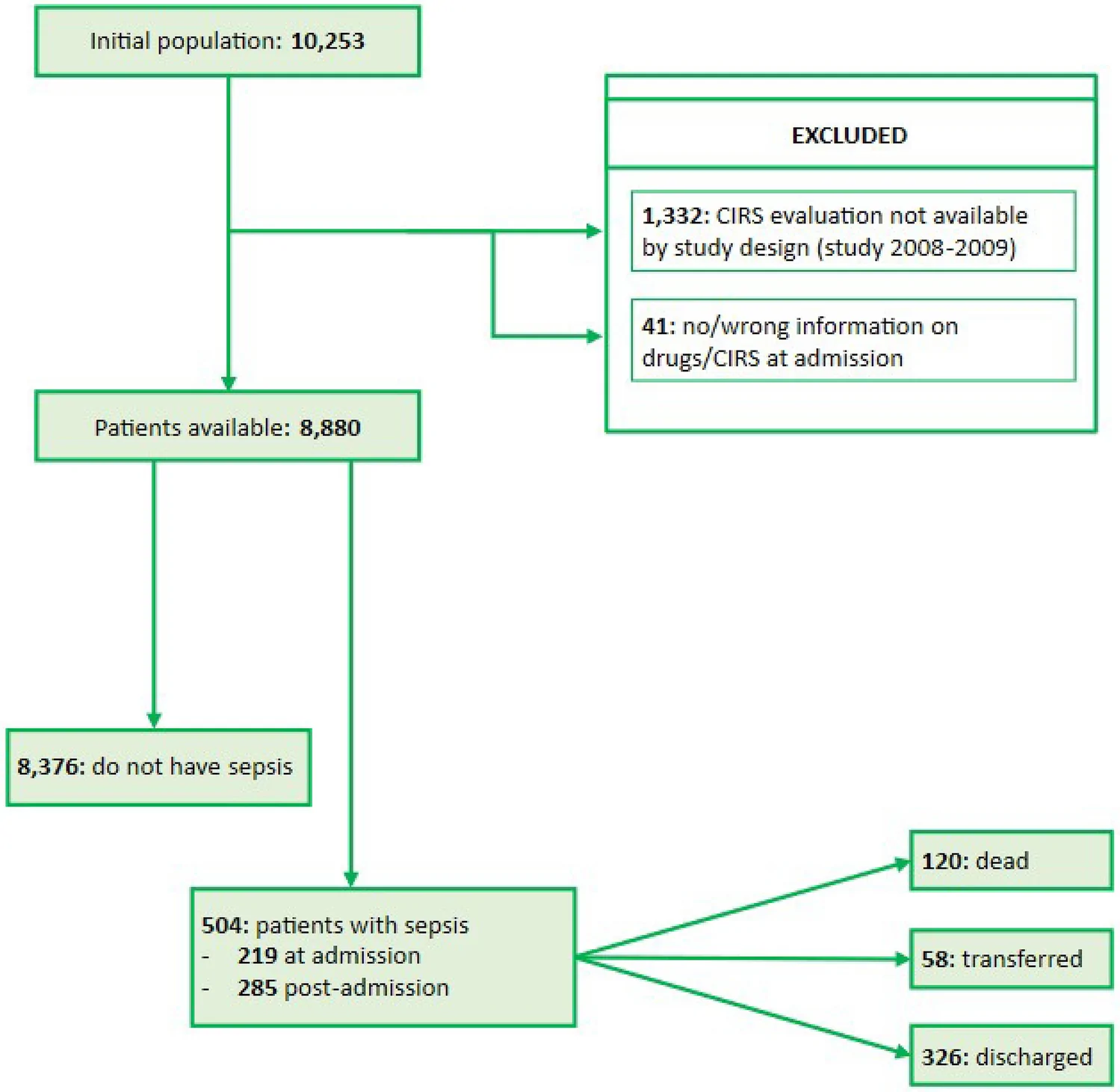
Flow-chart of the population selection

For the purposes of this analysis, cases enrolled in the 2008 edition of the registry (n=1332) were excluded, as the Cumulative Illness Rating Scale (CIRS) was not collected on that year by study design. Additionally, patients with missing, inconsistent, or clearly erroneous data regarding either CIRS scores or medication use at the time of hospital admission were excluded (n=41). Thus, the final study cohort consisted of 8880 individuals (Fig. 1).

Inclusion in the sepsis group required a documented sepsis diagnosis from at least one of the following sources: reason for hospitalization, admission diagnoses, indication for drug therapy, intercurrent clinical events during hospitalization, or cause of death. Sepsis cases were identified using ICD-9 codes (Supplementary Table S1). Additional registry labels for severe sepsis and septic shock were extracted.

Baseline comorbidities were defined using ICD-9 codes as follows: malignancy (140–208), chronic respiratory disease (491, 493, 515, 518), chronic heart disease and hypertensive heart and renal disease (402, 404, 428), chronic kidney disease (585), diabetes mellitus (250), chronic liver disease (571, 070), and neuropsychiatric disorders (433, 434, 438, 348, 290, 291, 340, 335, 357, 332).

Antimicrobial treatment for sepsis was classified according to ATC groups J01 and J02, and vasoactive agents [adrenaline (C01CA24), noradrenaline (C01CA03)] and dopamine (C01CA04) were also considered (Supplementary Table S2).

To further characterize sepsis, the most likely source of infection was identified by reviewing multiple records within the REPOSI registry and classified into predefined categories, including lower respiratory tract, urinary tract, intra-abdominal, gallbladder, skin or soft tissue, septic arthritis, cardiac or unknown origin, based on corresponding ICD-9 codes (Supplementary Table S1). Because of the structure of the REPOSI registry, catheter-related bloodstream infections (CRBSI) and other device-associated infections could not be identified as distinct causes of sepsis.

### Statistical analysis

Patients baseline characteristics, drug therapy, comorbidities and clinical parameters at admission were described using frequencies and percentages for categorical variables, and medians with interquartile ranges for continuous ones. Group differences were assessed using chi-square tests for categorical variables and Wilcoxon rank-sum tests for continuous ones and reporting p-values.

The primary aim of the study was to evaluate factors associated with in-hospital mortality in older adults with clinically recognized sepsis admitted to Internal Medicine wards. Secondary analyses compared baseline characteristics between septic and non-septic cases and assessed mortality determinants according to sepsis onset setting (community- vs hospital-acquired). We also explored prolonged length of stay (LOS), measured as days from admission to discharge or death dichotomized according to the median value, but only for cases of in sepsis diagnosed at admission, because LOS in hospital-acquired sepsis may reflect hospitalization preceding sepsis onset. Additional descriptive analyses assessed geographic distribution, infection sources and treatment patterns.

Latent Class Analysis (LCA) was applied to identify homogeneous multimorbidity subgroupsamong patients presenting with at least two chronic conditions, which were compared with those with no/low comorbidity. LCA is a probabilistic, model-based clustering technique that assigns individuals to mutually exclusive groups according to the similarity of their response patterns across observed variables [19]. The model included seven chronic conditions recorded at admission: malignancy, chronic respiratory disease, chronic heart failure, hypertensive heart and renal disease, chronic kidney disease, chronic liver disease, neuropsychiatric disorders and diabetes mellitus. The analysis included 229 patients presenting two or more of these comorbidities, whereas patients with none or one comorbidity (n=275) were grouped into a “*No/Low Comorbidity category*” and used as the reference group in subsequent analyses.

The optimal number of classes was determined by balancing statistical fit and clinical interpretability, using the Bayesian Information Criterion (BIC) and the Akaike Information Criterion (AIC). Class assignment and characterization were based on posterior probabilities, observed-to-expected ratios (O/E), and exclusivity measures (Supplementary Tables S7-S8).

Class composition was assessed using O/E ratios and exclusivity, which reflect the relative overrepresentation of conditions within each class. Conditions were considered part of a class pattern if the O/E ratio was ≥ 1.5 or exclusivity ≥ 25%, and the latent classes were named accordingly.

The latent classes identified in the final model were used to create a categorical variable, which was included as a predictor in subsequent analyses. Participants with 0–1 comorbidity, who were not included in the LCA, were considered as the reference group to allow comparison with multimorbid patients.

Associations between variables and outcomes were first explored using univariate logistic regression. Multivariable models were adjusted for age and sex, and then for relevant clinical covariates: Barthel index, serum albumin, serum glucose, body mass index (BMI), anticholinergic drug cognitive burden (ACB) index, admission year (2010–2016 vs. 2017–2023), and LCA class membership [20]. For each model, odds ratio (OR), 95% confidence intervals (CIs) and p-values were reported. To improve interpretability, selected continuous variables were rescaled before inclusion in regression models: glucose was modelled per 10-mg/dl increase and Barthel Index per 10-point increase. Missing data were handled through multiple imputation, performed using chained equations applied to albumin, glucose, BMI and Barthel index, with the goal to preserve statistical power given the moderate sample size.

All analyses were performed using Stata/IC v15.1 (StataCorp, College Station, TX).

## Results

### General characteristics of the study population

Among the 8880 patients included in the study, 504 (5.7%) had a diagnosis of sepsis: 219 cases (43.5%) were community-acquired, while the majority (285 cases, 56.5%) developed sepsis during hospitalization (Fig. 1). Differences according to sepsis setting onset were observed. Hospital-acquired sepsis was associated with higher in-hospital mortality compared with community-acquired sepsis, respectively 36.1% vs. 14.9% (Supplementary Tables S9–S10). In addition, multimorbidity patterns showed differential prognostic relevance according to the sepsis onset setting. The chronic multimorbidity profile identified by LCA (class 2) was independently associated with in-hospital mortality in community-acquired sepsis, whereas no significant association was observed in hospital-acquired cases (Supplementary Tables S9–S10).

Within the sepsis cohort, 51 patients had a documented diagnosis of septic shock and 95 of severe sepsis according to the registry coding in use during the study period.

Table 1 reports the characteristics of the study population, stratified by the presence or absence of sepsis. Patients with sepsis were, on average, older, with lower Barthel Index scores, higher clinical frailty scores, greater comorbidity burden at admission, were more frequently admitted from residential facilities rather than home and were often previously hospitalized within the previous 12 months. In addition, patients with sepsis more frequently exhibited hypoalbuminemia, neuropsychiatric and neoplastic diseases, greater need for urinary catheterization during hospitalization, and had a longer length of stay. Geographical and temporal analyses revealed a relatively higher prevalence of sepsis in Northern regions and during the 2017–2023 period (Table 1).

**Table 1:** Demographic and clinical characteristics of patients with and without sepsis (N = 8880)

Variable	No Sepsis (n = 8376)	Sepsis (n = 504)	p-value^a^
Age *(years), median (IQR)*	80 (74–85)	82 (75–87)	<0.001
Gender, *n (%)*			0.27
Female	4234 (50.5)	242 (48.0)	
Male	4142 (49.5)	262 (52.0)	
Length of Stay *(days), median (IQR)*	9 (6–14)	14 (8–22)	<0.001
Origin, *n (%)*			<0.001
Home	7811 (94.4)	440 (88.5)	
Nursing home	237 (2.9)	37 (7.4)	
Other (specify)	222 (2.7)	20 (4.0)	
Ward, *n (%)*			0.22
Geriatrics	1624 (19.4)	109 (21.6)	
Internal Medicine	6,752 (80.6)	395 (78.4)	
Region, *n (%)*			0.002
North	3793 (45.3)	268 (53.2)	
Center	2268 (27.1)	113 (22.4)	
South and Islands	2315 (27.6)	123 (24.4)	
Previous 12 months hospital admissions, *n (%)*	2841 (34.0)	232 (46.1)	<0.001
Frailty index, *median (IQR)*	7.5 (5.8–10)	9.5 (7–12.5)	<0.001
Barthel index before admission, *median (IQR)*	91 (68–100)	73 (31–96)	<0.001
Albumin *(g/dl), median (IQR)*	3.4 (3.0–3.8)	3.0 (2.5–3.3)	<0.001
Glucose *(mg/dl), median (IQR)*	108 (91–138)	113 (92–144)	0.065
Body Mass Index (BMI) *(kg/m*^*2*^*), median (IQR)*	25.35 (22.58–28.44)	24.61 (22.10–27.68)	0.004
ACB index, *median (IQR)*	10 (0–22)	14 (0–29)	<0.001
Malignancy, *n (%)*	1194 (14.3)	113 (22.4)	<0.001
Chronic Respiratory disease, *n (%)*	711 (8.5)	34 (6.7)	0.17
Heart failure, *n (%)*	2561 (30.6)	152 (30.2)	0.84
Renal diseases, *n (%)*	1815 (21.7)	134 (26.6)	0.01
Diabetes, *n (%)*	2,302 (27.5)	131 (26.0)	0.47
Chronic liver diseases, *n (%)*	821 (9.8)	43 (8.5)	0.35
Neuropsychiatric diseases, *n (%)*	1913 (22.8)	161 (31.9)	<0.001
CIRS severity at admission, *median (IQR)*	1.62 (1.38–1.85)	1.69 (1.46–1.92)	<0.001
CIRS comorbidity at admission, *median (IQR)*	3 (2–4)	3 (2–4)	0.001
N. of drugs, *median (IQR)*	5 (4–8)	6 (4–8)	0.80
Polytherapy, *n (%)*	5242 (62.6)	322 (63.9)	0.56
Urinary catheter at admission, *n (%)*			<0.001
No	7848 (95.4)	423 (85.8)	
Yes	381 (4.6)	70 (14.2)	
Admission Year, *n (%)*			<0.001
2010–2016	4504 (53.8)	161 (31.9)	
2017–2023	3872 (46.2)	343 (68.1)	

Data are presented as number (percentage) [n (%)] for categorical variables or median (interquartile range) [median (IQR)] for continuous variables unless otherwise specified

^a^ p-values were obtained using the Wilcoxon rank-sum test for continuous variables and the chi-square test for categorical variables

*BMI* Body Mass Index, *IQR* Interquartile Range, *CIRS* Cumulative Illness Rating Scale, *ACB* Anticholinergic Cognitive Burden

Missing values: Length of stay (n=20); Origin (n=113); Previous hospital admissions (n=9); Barthel Index (n=1,528); Albumin (n=3,208); Glucose (n=460); BMI (n=1,232); CIRS (n=14); Urinary catheter (n=158)

### Latent class analysis (LCA)

LCA identified two multimorbidity patterns with at least two selected chronic conditions. The first, *Oncologic–Hepatologic*, was characterized by a high prevalence of malignancy and chronic liver diseases with limited co-occurrence of other chronic conditions. The second, *Chronic Multimorbidity*, included patients with predominant chronic respiratory disorders frequently coexisting with cardio-renal-metabolic and neuropsychiatric comorbidities. Patients with no/low comorbidity were retained as the reference group.

Further details are provided in Supplementary Table S7-S8.

### Mortality

In-hospital mortality was higher in patients with sepsis than in non-septic cases (23.8% vs 3.0%; Table 1). Among patients with sepsis with available discharge outcome, mortality differed markedly according to sepsis onset setting, being more than twice in sepsis developed during hospitalization than in sepsis already present ad admission (36.1% vs. 14.9%; 91/252 vs. 29/194). Notably, the excess in-hospital mortality observed among patients with sepsis was largely driven by hospital-acquired episodes, which were associated with a substantially higher risk of death compared with community-acquired sepsis.

In the multivariate analysis (Table 2), three admission variables were independently associated with  in-hospital mortality among patients with sepsis: advanced age (OR 1.05; 95% CI 1.02–1.09; *p* = 0.001), lower Barthel Index (OR 0.90 per 10-point increase; 95% CI 0.90–1.00; *p* = 0.02), and lower albumin levels (OR 0.59 per 1 g/dL increase in serum albumin; 95% CI 0.36–0.97; *p* = 0.043). A borderline association with lower in-hospital mortality was also observed for admissions  during 2017-2023 compared with 2010-2016 (OR 0.61; 95% CI 0.37-0.99; p = 0.05).

**Table 2:** Association of patient characteristics with in-hospital mortality in sepsis: logistic regression model (N = 446)

Variable	Discharged (n = 326)	Dead (n = 120)	Univariate OR (95% CI)	Multivariate OR (95% CI)	p-value ^a^
Age *(years), mean (*± *SE)*	80.1 (0.4)	83.7 (0.7)	1.06 (1.03–1.10)	1.05 (1.02–1.09)	0.001***
Barthel index *(per 10-point increase), mean (*± *SE)*	66.1 (2.0)	49.5 (3.7)	0.90 (0.82–0.90)	0.90 (0.90–1.00)	0.02*
Albumin *(g/dl), mean (*± *SE)*	3.0 (0.1)	2.8 (0.1)	0.49 (0.32–0.76)	0.59 (0.36–0.97)	0.04*
Glucose *(per 10 mg/dL increase), mean (*± *SE)*	131.1 (4.2)	131.9 (6.9)	1.00 (1.00–1.00)	1.00 (1.00–1.00)	0.99
BMI *(kg/m*^*2*^*), mean (*± *SE)*	25.1 (0.3)	24.4 (0.5)	0.97 (0.92–1.02)	0.989 (0.94–1.05)	0.76
ACB index*, mean (*± *SE)*	20.7 (1.5)	27.3 (2.8)	1.01 (1.00–1.01)	1.01 (1.00–1.01)	0.06
Gender, *n (%)*					
Female (Ref.)	151 (46.3)	59 (49.2)	Ref	Ref	0.66
Male	175 (53.7)	61 (50.8)	0.89 (0.59–1.36)	1.07 (0.67–1.68)	
Admission Year, *n (%)*					
2010–2016 (Ref.)	103 (31.6)	45 (37.5)	Ref	Ref	0.05*
2017–2023	223 (68.4)	75 (62.5)	0.77 (0.50–1.19)	0.61 (0.37–0.99)	
LCA class, *n (%)*					
No/Low comorbidity (Ref.)	189 (58.0)	52 (43.3)	Ref	Ref	0.07
Oncologic–liver disease	45 (13.8)	21 (17.5)	1.70 (0.93–3.10)	2.01 (1.05–3.85)	
Chronic multimorbidity with respiratory involvement	92 (28.2)	47 (39.2)	1.86 (1.16–2.96)	1.51 (0.91–2.54)	

All models were developed using Multiple Imputation to handle missing data for Barthel Index, Albumin, Glucose and BMI

Continuous variables are presented as mean (standard error) and categorical variables are expressed as frequencies (percentage). Odds ratios refer to a one-unit increase unless otherwise specified; for glucose, odds ratios refer to a 10 mg/dL increase; for the Barthel Index to a 10-point increase

Global p-value of the multivariate model (chi-square test): <0.0001

*SE* Standard Error, *OR* Odds Ratio, *CI* Confidence Interval, *LCA* Latent Class Analysis, *BMI* Body Mass Index, *ACB* Anticholinergic Cognitive Burden, Ref. Reference Category

^a^ p-value refers to the multivariate analysis: ^*^ *p* < 0.05, ^**^ *p* ≤ 0.01, ^***^ *p* ≤ 0.001

The analysis includes 446 septic patients; 58 patients transferred to other wards or hospitals were excluded due to unavailable in-hospital outcome

When analyses were stratified by sepsis onset setting, different mortality patterns emerged: in community-acquired sepsis, age and Barthel Index remained significant predictors of mortality, in line with the overall septic cohort, whereas serum albumin levels were not (Supplementary Table S9). In this subgroup, multimorbidity phenotypes identified by LCA were variably associated with mortality: compared with the reference group, the chronic multimorbidity class was associated with higher odds of death (OR 4.34; 95% CI 1.45–12.96; p = 0.03), whereas cases in the oncologic–hepatologic class showed no statistically significant increase in mortality (OR 2.78; 95% CI 0.64–12.05). These associations were not observed in the overall septic cohort (Table 2). In contrast, in hospital-acquired sepsis, age was the only variable independently associated with in-hospital mortality (Supplementary Table S10).

### Length of hospital stay (LOS)

Median LOS was longer among patients with sepsis than in non-septic cases (14 days [IQR 8–22] vs. 9 days [IQR 6–14], *p* < 0.001) (Table 1). Because the exact timing of sepsis onset was unavailable for hospital-acquired cases, LOS analyses were considered to be more appropriately interpretable in community-acquired sepsis. In this subgroup, prolonged LOS was independently associated with a higher anticholinergic drug burden (OR 1.02 per one-point increase; 95% CI 1.00–1.03; *p* = 0.013), while age, Barthel Index score, serum albumin levels and LCA class were not (Supplementary Table S11).

### Source of sepsis

Source identification differed between community-acquired and hospital-acquired sepsis, being available in 42% and 67% of cases, respectively (Supplementary Tables S3-S4). In both settings, lower respiratory tract infections were the most common source (32 and 29%), followed by urinary (16 and 18%), intra-abdominal (15% in both), and hepatobiliary infections. Hospital-acquired sepsis showed a broader spectrum of sources, including skin and soft tissue, as well as cardiac infections.

### Pharmacological therapies

Across the study period, the most frequently used antimicrobials were β-lactam/β-lactamase inhibitor combinations, followed by third-generation cephalosporins, carbapenems, glycopeptides, and fluoroquinolones; triazoles/tetrazoles predominated among antifungals. Between 2010–2016 and 2017–2023 the use of carbapenems and fluoroquinolones declined, while prescriptions of other antibacterial agents (e.g., linezolid, daptomycin, fosfomycin) increased moderately (Supplementary Tables S5a-S5b). Vasoactive amines were rarely employed (Supplementary Tables S5a-S5b-S6).

## Discussion

Older adults with clinically recognized sepsis admitted to internal medicine and geriatric wards represent a clinically complex population at high risk of adverse outcomes. While research on sepsis has predominantly focused on ICU, its burden and determinants in internal medicine wards remain comparatively underexplored, despite substantial mortality, disability, and prolonged hospital stay [31]. Using data from the REPOSI registry, this retrospective study provides a real-world description of sepsis burden and outcomes, including in-hospital mortality and length of stay [3].

### Sepsis burden in internal medicine wards

The prevalence of clinically recognized sepsis in our cohort (5.7%) aligns with previous European studies conducted in internal medicine wards, with reported rates ranging between 4 and 6% [21–23]. The higher prevalence observed in 2017–2023 likely reflects the increased awareness of sepsis  following the introduction of Sepsis-3 criteria. The higher prevalence recorded in Northern Italy may reflect regional demographic differences, participating centres distribution, local healthcare organization and patient case-mix, and temporal factors including the COVID-19 pandemic. More than half of sepsis cases developed during hospitalization, underscoring hospital-acquired sepsis as a major clinical problem in medical wards, where it was associated with prolonged hospitalization and substantially higher mortality than community-acquired sepsis.

Distinct predictors of in-hospital mortality emerged according to the sepsis onset setting. Community-acquired sepsis appeared associated with age, functional impairment and multimorbidity phenotype, whereas age remained the predominant determinant of in-hospital mortality in hospital-acquired sepsis.

### Clinical vulnerability, frailty and multimorbidity

Compared with non-septic patients, those with sepsis exhibited greater frailty, multimorbidity, functional impairment and reduced physiological reserve [15, 24]. Advanced age emerged as the strongest independent predictor of in-hospital mortality, confirming its central prognostic role in older adults with sepsis. Cancer-related immunosuppression and healthcare exposure may increase susceptibility to infection and poor outcomes, while neuropsychiatric disorders may contribute through functional impairment, delayed symptom recognition and anticholinergic drug burden [25, 26]. Taken together, these observations suggest that, in older adults, ward-managed sepsis frequently occurs at the intersection of infection and pre-existing vulnerability [27].

The application of LCA identified two main classes: an oncologic–hepatologic profile and a chronic multimorbidity profile characterized by cardio–renal–metabolic, respiratory, and neuropsychiatric diseases. These classes should be interpreted as statistically derived, context-dependent constructs rather than definite biological endotypes. Their composition reflects the variables available within the registry and may differ in other populations. Nevertheless, they provide a clinically meaningful framework for characterizing vulnerability heterogeneity and may complement traditional risk stratification approaches. Comparable applications of LCA have been previously reported in sepsis research, both in acute cohorts and among sepsis survivors [28, 29].

### Determinants of in-hospital mortality

Overall in-hospital mortality among patients with sepsis was high (23.8%), consistently with the upper range reported in European cohorts (15–25%), and was markedly higher in hospital-acquired sepsis (36.1%) [4, 41]. This estimate is also consistent with the pooled 30-day mortality of 23.6% reported for European studies in a large international systematic review and meta-analysis [30]. Our findings complement the limited evidence from internal medicine and general ward cohorts, where sepsis is associated with substantial mortality and remains considerably less studied than in emergency department and ICU populations [22].

Advanced age, functional impairment, and hypoalbuminemia confirmed the prognostic relevance of baseline vulnerability in older adults with sepsis in the overall cohort. Lower levels of albuminemia should be interpreted as a marker of vulnerability rather than a therapeutic target, as its prognostic value does not imply benefit from albumin supplementation [32]. No consistent associations were observed between mortality and sex, BMI, or glycemic levels, in line with findings in the literature [33].

These findings should be interpreted in the light of the structure of the REPOSI registry, which was not specifically designed to capture acute physiological derangement at the time of sepsis onset. As a result, the observed associations primarily reflect baseline clinical vulnerability rather than acute illness severity. This interpretation should not imply that multimorbidity, functional impairment, or nutritional status outweigh acute illness severity in determining prognosis. Rather, acute severity and early management could not be adequately assessed within the present dataset. Therefore, our findings identify prognostic factors related to those of baseline clinical vulnerability which were observable in the frame of this ward-based population [34].

Distinct prognostic associations emerged according to sepsis onset. In community-acquired sepsis, mortality was associated with the chronic multimorbidity phenotype, whereas from the association for the oncologic–hepatologic class, a trend did not reach statistical significance. In line with large national cohort studies indicating that multimorbidity is associated with increased risk of sepsis and higher in-hospital mortality, our findings suggest that multimorbidity may represent a marker of biological vulnerability rather than a mere clinical background [35]. Although advanced cancer and severe liver disease are established states of immune vulnerability, the broader chronic multimorbidity phenotype identified in the present cohort showed a stronger association with mortality in community-acquired sepsis [36, 37]. These observations suggest that specific combinations of chronic conditions, rather than their number alone, may shape prognosis in community-acquired sepsis. However, the direction of the association remained consistent with an increased mortality risk in the oncologic–hepatologic phenotype, although the lack of statistical significance may reflect the heterogeneity of this class, preferential referral of high-risk patients to specialized units, and limited statistical power owing to small sample size.

In contrast, in hospital-acquired sepsis, age remained the sole independent predictor of mortality, while baseline vulnerability markers did not retain independent prognostic significance. This may reflect the influence of time-dependent care processes, including early administration of proper antimicrobial therapy, prompt source identification and control, escalation of care including ICU transfer: factors that are not measured by the Registry, making the prognostic contribution of baseline vulnerability parameters less apparent [38]. Although SOFA scores were unavailable, exploratory analyses showed substantially higher mortality among patients with clinically documented severe sepsis/septic shock (41.5% vs. 17.3%), supporting the contribution of acute illness severity to prognosis. Residual confounding due to unmeasured severity variables (eg. lactate levels, full SOFA score) cannot be excluded.

### Determinants of prolonged hospitalization

Median LOS was longer among patients with sepsis than non-septic cases (14 vs. 9 days), consistently with medical ward-based international studies [2]. Because the exact timing of sepsis onset was unavailable in hospital-acquired cases, LOS analyses were considered more interpretable in community-acquired sepsis. In this subgroup, prolonged LOS (> 14 days) was independently associated with higher anticholinergic drug burden (ACB index). Higher anticholinergic burden may contribute to longer hospitalization through increased risk of delirium, cognitive decline, falls, and functional deterioration. As such, it may represent a potentially modifiable target in hospitalized patients [39].

### Infection sources and treatment patterns

Lower respiratory tract infections were the most frequent source of sepsis, followed by urinary tract, intra-abdominal, and hepatobiliary infections, in line with large international cohorts [5, 40, 41]. Older adults are particularly susceptible to respiratory and urinary infections because of age-related vulnerability and multimorbidity [42, 43]. Source identification differed between community and hospital-acquired sepsis. In community-acquired cases, the source remained unidentified in more than half of patients, likely reflecting atypical presentations, delayed and limited access to early diagnostic investigations [44]. In hospital-acquired sepsis, respiratory and urinary infections remained prevalent, but a broader spectrum of sources was identified, including skin, soft-tissue, and cardiac infections, consistent with healthcare-related exposures.

Antimicrobial therapy mainly relied on broad-spectrum antibiotics, such as β-lactams, cephalosporins, and carbapenems, consistent with international recommendations for initial sepsis management [45, 46]. Over time, fluoroquinolone, cephalosporin and antifungal drugs use declined, while more targeted agents such as fosfomycin, linezolid, and daptomycin use increased [47]. Despite the trend towards appropriateness, carbapenem use remained substantial even in community acquired sepsis, possibly reflecting concerns about multidrug resistant organisms in multimorbid patients. Antimicrobial treatment patterns were described for the overall as well as community-acquired sepsis cohorts, while analyses of hospital-acquired sepsis were limited by treatment changes during hospitalization. Vasopressor use was rare, reflecting the ward-based setting, where septic shock prompts ICU referral [45]. Increased noradrenaline use likely reflects evolving adherence to sepsis guidelines. These findings align with real-world studies from non-ICU settings, where vasopressors are used in fewer than 3% of cases and highlight differences from critical care settings [48].

### Clinical implications and future directions

These findings indicate that, in internal medicine wards, sepsis may reflect the convergence of infection with multimorbidity, functional impairment, nutritional compromise, and pharmacological burden. LCA-derived multimorbidity patterns may help to identify subgroups of patients at differing risk of mortality and prolonged hospitalization. Integrating functional status, nutritional markers, and medication review into routine clinical assessment may improve risk stratification. Future studies should validate multimorbidity phenotypes in other cohorts, incorporate acute severity measures, and explore biological correlates of vulnerability in order to advance precision medicine approaches in geriatric sepsis.

### Strengths and limitations

The main strengths of this study include its nationwide multicentre setting, large sample size, prolonged observation period, and real-world representation of internal medicine and geriatric wards across Italy. The study is based on a retrospective analysis of prospectively collected data from the REPOSI registry, where consecutively enrolled patients are evaluated according to a standardized protocol using predefined clinical variables across participating centres. These design characteristics reduce selection and information bias, strengthen external validity, and provide a robust framework for investigating clinically recognised sepsis in routine internal medicine practice. The internal consistency of the identified sepsis cohort is further supported by the observed prevalence, antimicrobial treatment patterns and mortality, which are consistent with previous internal medicine cohorts. The study addresses an underexplored clinical setting, as most sepsis literature stems from ICU populations despite the growing burden of sepsis among older patients admitted to medical wards. This is particularly relevant given the distinctive biological and clinical features of older adults. The integration of functional, nutritional, pharmacological and multimorbidity-related variables together with the application of an innovative multimorbidity-based approach provides novel clinical insights.

Limitations include the retrospective design, possible sepsis misclassification related to ICD-9 coding, and the lack of availability of key acute-severity variables (e.g., CRP, lactate values, dynamic severity variables, full SOFA score), which limited Sepsis-3 adjudication and comparison of illness severity with ICU-based sepsis cohorts. Although several laboratory and physiological variables were available, they were generally recorded at ward admission rather than at sepsis onset and therefore could not reliably reflect the acute severity of the septic episode. Because REPOSI spans 2008–2023 and was designed to investigate a broad spectrum of medical conditions rather than any single disease or syndrome, it does not systematically collect all condition-specific variables required for retrospective diagnostic adjudication, including all SOFA components. Accordingly, sepsis identification relied on clinician-assigned ICD-9 coding retrieved from multiple predefined registry sections, rather than on hospital discharge codes alone, a strategy widely used in large epidemiological studies, but inherently liable to some degree of misclassification. As with other nationwide prospective registries, retrospective adjudication of every recorded diagnosis according to condition-specific diagnostic criteria was beyond the scope of the registry design. Consequently, the present findings should be interpreted as referring to clinically recognised sepsis rather than retrospectively adjudicated Sepsis-3-defined sepsis.

Similarly, the available dataset did not provide sufficient information to identify catheter-related bloodstream infections (CRBSI) or other device-associated infections as distinct etiological categories, preventing a more detailed characterization of hospital-acquired sepsis [49]. Nevertheless, exploratory analyses showed higher mortality in clinician-documented severe sepsis/septic shock, consistently with the prognostic role of acute severity.

The REPOSI registry includes only patients admitted to internal medicine and geriatric wards, while patients with more severe acute presentations may be managed in intensive care units, whereas those with specific infectious or oncologic conditions and a lower burden of comorbidity may be preferentially admitted to specialized units. This setting-related selection may have influenced the case mix and the distribution of multimorbidity profiles, limiting generalizability of the identified multimorbidity phenotypes.

Finally, residual confounding, particularly in hospital-acquired sepsis, cannot be excluded because data on acute management were not available, including devices, fluid resuscitation, time-to-antibiotics, antibiotic appropriateness, and adequacy of hemodynamic support. In hospital-acquired sepsis, the exact timing of onset was unavailable, therefore, LOS may partly reflect hospitalization preceding sepsis onset rather than the sole impact of sepsis itself. Despite these limitations, the study provides relevant real-world insights into sepsis among elderly multimorbid patients admitted to internal medicine and geriatric wards.

## Conclusion

In older adults admitted to internal medicine wards, sepsis may be understood and managed not merely as an acute infectious syndrome but as the clinical expression of an underlying vulnerability shaped by age, multimorbidity and treatment burden. Integrating geriatric dimensions into sepsis assessment may help identify high-risk patients and support more personalized management strategies.

## Supplementary Information

Below is the link to the electronic supplementary material.

Supplementary file1 (DOCX 47 KB)

## Data Availability

The datasets used and analyzed during the current study are available from the corresponding author on reasonable request.
