# Supplementary material for: Clinically recognized sepsis in older adults admitted to medical wards: patient characteristics and hospital outcomes from the REPOSI register
Source: Intern Emerg Med. 2026 Aug 24;21(8):2951–64. doi: 10.1007/s11739-026-04501-z (PMC13751800; doi:10.1007/s11739-026-04501-z)
Supplement: Supplementary file 1 [file 11739_2026_4501_MOESM1_ESM.docx]

**Supplementary Table S1** Diagnostic ICD-9-CM codes used to identify sepsis and sources of infection.

| **Diagnosis** | **ICD-9 codes** |
| --- | --- |
| ***Sepsis*** |  |
| Salmonella septicemia | 003.1 |
| Anthrax septicemia | 022.3 |
| Streptococcal septicemia | 038.0 |
| Staphylococcal septicemia, unspecified | 038.10 |
| Methicillin susceptible Staphylococcus aureus septicemia | 038.11 |
| Other staphylococcal septicemia | 038.19 |
| Pneumococcal septicemia | 038.2 |
| Septicemia due to anaerobes | 038.3 |
| Septicemia due to other gram-negative organisms | 038.4x |
| Other specified septicemias | 038.8 |
| Unspecified septicemia | 038.9 |
| Herpetic septicemia | 054.5 |
| Septic shock | 785.52 |
| Sepsis | 995.91 |
| Severe sepsis | 995.92 |
| ***Source of infection*** |  |
| Lower respiratory tract infection | 480.x, 481, 482.xx, 483.x, 484.x, 485, 487.x, 003.2x, 006.4, 491.2x, 507.0, 510.x, 511.0, 511.1, 513.x |
| Other origin | 519.0x, 530.8x, 536.4x, 569.6x, 996.6x, 998.5x, 997.6x, 707.0x, V58.8x |
| Urinary tract infection | 954.x, 098.0, 098.1x, 098.2, 098.3x, 099.4x, 599.0, 590.2, 590.3, 590.8x, 590.9, 601.0, 601.2, 616.0, 639.0, 995.6x |
| Intra-abdominal infection | 007.x, 540.x, 541, 542, 567.xx, 008.xx, 009.x, 006.0, 006.1, 006.2, 006.3, 006.9, 562.xx, 569.5, 614.5 |
| Gallbladder infection | 572.0, 574.0x, 574.3x, 575.0, 575.1x, 575.4, 576.1, 576.3, 576.4 |
| Unknown origin | 024, 027.8, 036.3, 036.4x, 036.8x, 036.9, 041.0x, 041.1x, 041.2, 041.4x, 041.5, 041.6, 041.7 |
| Multidrug-resistant organism-related infection | V09 |
| Skin or soft tissue infection | 035, 040.0, 376.0x, 478.2x, 528.3, 567.3x, 728.8x |
| Septic arthritis | 323.xx, 711.0x, 711.9x, 985.0 |
| Heart infection (endocarditis, myocarditis) | 421.x, 032.8x, 036.4x, 093.2x, 093.8x, 098.8x, 112.8x, 115.0x, 115.9x, 130.3, 391.1, 391.2, 422.9x, 513.1 |
| Fungal infection | 039.8, 039.9, 112.4, 112.5, 112.8x, 117.3, 115.9x, 117.9, 136.3 |

**Supplementary Table S2** ATC codes used to identify pharmacological treatments.

| **Treatment** | **ATC codes** |
| --- | --- |
| Tetracyclines | J01AA |
| Penicillin with extended spectrum | J01CA |
| Combinations of penicillin, incl. beta-lactamase inhibitors | J01CR |
| Third-generation cephalosporins | J01DD |
| Fourth-generation cephalosporins | J01DE |
| Carbapenems | J01DH |
| Combinations of sulfonamides and trimethoprim, incl. derivatives | J01EE |
| Macrolides | J01FA |
| Lincosamides | J01FF |
| Other aminoglycosides | J01GB |
| Fluoroquinolones | J01MA |
| Glycopeptide antibacterials | J01XA |
| Polymyxins | J01XB |
| Imidazole derivatives | J01XD |
| Other antibacterials | J01XX |
| Triazole and tetrazole derivatives | J02AC |
| Other antimycotics for systemic use | J02AX |
| Noradrenaline | C01CA03 |
| Dopamine | C01CA04 |
| Adrenaline | C01CA24 |

**Supplementary Table S3** Proportion of patients identified at admission according to sepsis origin (N = 219).

| **Source of infection** |  | **Frequency (n)** | **Percentage ^a^ (%)** |
| --- | --- | --- | --- |
| Lower respiratory tract infection |  | 30 | 32.6 |
| Other origin |  | 24 | 26.1 |
| Urinary tract infection |  | 15 | 16.3 |
| Intra-abdominal infection |  | 14 | 15.2 |
| Gallbladder infection |  | 5 | 5.4 |
| Unknown origin |  | 2 | 2.2 |
| Septic arthritis |  | 1 | 1.1 |
| Fungal infection |  | 1 | 1.1 |
| *Total with available information* |  | *92* | *100* |

Data are presented as absolute numbers (n) and percentages (%).

^a^ Percentages are calculated based on the number of patients with available information (n = 92).

Missing data: information on the source of infection was not available for 127 patients (58% of the sepsis cohort identified at hospital admission).

**Supplementary Table S4** Proportion of patients identified during hospital stay according to sepsis origin (N = 285).

| **Source of infection** | **Frequency (n)** | **Percentage ^a^ (%)** |
| --- | --- | --- |
| Lower respiratory tract infection | 56 | 29.3 |
| Other origin | 42 | 22.0 |
| Urinary tract infection | 34 | 17.8 |
| Intra-abdominal infection | 29 | 15.2 |
| Gallbladder infection | 17 | 8.9 |
| Unknown origin | 4 | 2.1 |
| Multidrug-resistant organism | 3 | 1.6 |
| Skin or soft tissue infection | 2 | 1.0 |
| Heart infection (endocarditis, myocarditis) | 2 | 1.0 |
| Fungal infection | 2 | 1.0 |
| *Total with available information* | *191* | *100* |

Data are presented as absolute numbers (n) and percentages (%).

^a^ Percentages are calculated based on the number of patients with available information (n = 191).

Missing data: information on the source of infection was not available for 94 patients (33% of the sepsis cohort identified at hospital admission).

**Supplementary Table S5a** Drug therapy for sepsis: active substance.

|  |  | **Admission Year** | |
| --- | --- | --- | --- |
| **Active substance** | **Overall (N = 504)** | **2010-2016 (n = 161)** | **2017-2023 (n = 343)** |
| J01 (Antibacterials for systemic use) | 468 (92.9) | 156 (96.9) | 312 (91.0) |
| J02 (Antimycotics for systemic use) | 58 (11.5) | 22 (13.7) | 36 (10.5) |

Data are presented as number (percentage) [n (%)].

**Supplementary Table S5b** Drug therapy for sepsis: therapeutic subclass.

|  |  | **Admission Year** | |
| --- | --- | --- | --- |
| **Therapeutic subclass** | **Overall (N = 504)** | **2010-2016 (n = 161)** | **2017-2023 (n = 343)** |
| J01AA (Tetracyclines) | 12 (2.4) | 2 (1.2) | 10 (2.9) |
| J01CA (Penicillin with extended spectrum) | 11 (2.2) | 5 (3.1) | 6 (1.7) |
| J01CR (Combinations of penicillin, incl. beta-lactamase inhibitors) | 243 (48.2) | 72 (44.7) | 171 (49.9) |
| J01DD (Third-generation cephalosporins) | 142 (28.2) | 54 (33.5) | 88 (25.7) |
| J01DE (Fourth-generation cephalosporins) | 7 (1.4) | 2 (1.2) | 5 (1.5) |
| J01DH (Carbapenems) | 137 (27.2) | 57 (35.4) | 80 (23.3) |
| J01EE (Combinations of sulfonamides and trimethoprim, incl. derivatives) | 19 (3.8) | 7 (4.3) | 12 (3.5) |
| J01FA (Macrolides) | 40 (7.9) | 15 (9.3) | 25 (7.3) |
| J01FF (Lincosamides) | 8 (1.6) | 1 (0.6) | 7 (2.0) |
| J01GB (Other aminoglycosides) | 22 (4.4) | 10 (6.2) | 12 (3.5) |
| J01MA (Fluoroquinolones) | 106 (21.0) | 59 (36.6) | 47 (13.7) |
| J01XA (Glycopeptide antibacterials) | 113 (22.4) | 44 (27.3) | 69 (20.1) |
| J01XB (Polymyxins) | 2 (0.4) | 1 (0.6) | 1 (0.3) |
| J01XD (Imidazole derivatives) | 33 (6.5) | 10 (6.2) | 23 (6.7) |
| J01XX (Other antibacterials) | 44 (8.7) | 6 (3.7) | 38 (11.1) |
| J02AC (Triazole and tetrazole derivatives) | 46 (9.1) | 18 (11.2) | 28 (8.2) |
| J02AX (Other antimycotics for systemic use) | 17 (3.4) | 6 (3.7) | 11 (3.2) |
| C01CA24 (Adrenaline) | 1 (0.2) | 1 (0.6) | 0 |
| C01CA03 (Noradrenaline) | 11 (2.2) | 1 (0.6) | 10 (2.9) |
| C01CA04 (Dopamine) | 19 (3.8) | 7 (4.3) | 12 (3.5) |

Data are presented as number (percentage) [n (%)].

**Supplementary Table S6** Drug therapy for sepsis diagnosed at admission: therapeutic subclass.

|  |  | **Admission Year** | | |
| --- | --- | --- | --- | --- |
| **Therapeutic subclass (%)** | **Overall**  **(N = 219)** | **2010-2016**  **(n = 60)** | **2017-2023**  **(n = 159)** |  |
| J01AA (Tetracyclines) | 5 (2.3) | 1 (1.7) | 4 (2.5) |  |
| J01CA (Penicillin with extended spectrum) | 6 (2.7) | 3 (5.0) | 3 (1.9) |  |
| J01CR (Combinations of penicillin, incl. beta-lactamase inhibitors) | 96 (43.8) | 26 (43.3) | 70 (44.0) |  |
| J01DD (Third-generation cephalosporins) | 59 (26.9) | 15 (25.0) | 44 (27.7) |  |
| J01DE (Fourth-generation cephalosporins) | 2 (0.9) | 1 (1.7) | 1 (0.6) |  |
| J01DH (Carbapenems) | 52 (23.7) | 20 (33.3) | 32 (20.1) |  |
| J01EE (Combinations of sulfonamides and trimethoprim, incl. derivatives) | 7 (3.2) | 0 | 7 (4.4) |  |
| J01FA (Macrolides) | 13 (5.9) | 2 (3.3) | 11 (6.9) |  |
| J01FF (Lincosamides) | 3 (1.4) | 0 | 3 (1.9) |  |
| J01GB (Other aminoglycosides) | 9 (4.1) | 2 (3.3) | 7 (4.4) |  |
| J01MA (Fluoroquinolones) | 45 (20.5) | 26 (43.3) | 19 (11.9) |  |
| J01XA (Glycopeptides antibacterial) | 36 (16.4) | 11 (18.3) | 25 (15.7) |  |
| J01XB (Polymyxins) | 2 (0.9) | 1 (1.7) | 1 (0.6) |  |
| J01XD (Imidazole derivatives) | 11 (5.0) | 4 (6.7) | 7 (4.4) |  |
| J01XX (Other antibacterial) | 14 (6.4) | 3 (5.0) | 11 (6.9) |  |
| J02AC (Triazole and tetrazole derivatives) | 19 (8.7) | 8 (13.3) | 11 (6.9) |  |
| J02AX (Other antimycotics for systemic use) | 8 (3.7) | 1 (1.7) | 7 (4.4) |  |
| C01CA24 (Adrenaline) | 1 (0.5) | 1 (1.7) | 0 |  |
| C01CA03 (Noradrenaline) | 5 (2.3) | 0 | 5 (3.1) |  |
| C01CA04 (Dopamine) | 6 (2.7) | 2 (3.3) | 4 (2.5) |  |

Data are presented as number (percentage) [n (%)].

**Supplementary Table S7** Prevalence of chronic diseases across Latent Class Analysis (LCA) groups.

| **Comorbidities** | **Class 1 (28%)** | **Class 2 (72%)** |
| --- | --- | --- |
| **Malignancy** | 1 | 0.06 |
| **Chronic Respiratory** **diseases** | 0 | 0.16 |
| **Chronic Heart failure** | 0.22 | 0.64 |
| **Chronic Kidney diseases** | 0.32 | 0.61 |
| **Diabetes mellitus** | 0.35 | 0.52 |
| **Chronic Liver diseases** | 0.19 | 0.1 |
| **Neuropsychiatric diseases** | 0.41 | 0.51 |

**Supplementary Table S8** Observed/expected (O/E) ratios and exclusivity of overrepresented diseases within clusters.

| **Comorbidities** | **Class 1 (28%)** | |  | **Class 2 (72%)** | | **Decision** |  |
| --- | --- | --- | --- | --- | --- | --- | --- |
|  | **O/E** | **Exclusivity** |  | **O/E** | **Exclusivity** |  |  |
| **Malignancy** | 3.09 | 0.87 |  | 0.18 | 0.13 | Class 1 | |
| **Chronic Respiratory** **diseases** | 0.00 | 0.00 |  | 1.39 | 1.00 | Class 2 | |
| **Chronic Heart failure** | 0.43 | 0.12 |  | 1.22 | 0.88 | Class 2 | |
| **Chronic Kidney diseases** | 0.61 | 0.17 |  | 1.15 | 0.83 | Class 2 | |
| **Diabetes mellitus** | 0.74 | 0.21 |  | 1.10 | 0.79 | Class 2 | |
| **Chronic Liver diseases** | 1.54 | 0.43 |  | 0.79 | 0.57 | Class 1 | |
| **Neuropsychiatric diseases** | 0.84 | 0.24 |  | 1.06 | 0.76 | Class 2 | |

Exclusivity represents the proportion of patients with the specific comorbidity belonging to a given LCA group.

**Supplementary Table S9** Association of patient characteristics with in-hospital mortality in sepsis diagnosed at admission: logistic regression model (N = 194).

|  | **Discharged**  **(n = 165)** | **Dead**  **(n = 29)** | **Univariate**  **OR (95% CI)** | **Age and sex adjusted**  **OR (95% CI)** | **Multivariate**  **OR (95% CI)** | **p-value ^a^** |
| --- | --- | --- | --- | --- | --- | --- |
| Age *(years), mean (±SE)* | 80.5 (0.4) | 85.0 (0.7) | 1.09 (1.03-1.16) | 1.10 (1.03-1.17) | 1.10 (1.02-1.19) | 0.01** |
| Barthel index *(per 10-point increase),* *mean (±SE)* | 64.3 (2.8) | 35.9 (6.9) | 0.82 (0.74-0.90) | 0.82 (0.74-0.90) | 0.82 (0.74-1.00) | 0.01** |
| Albumin *(g/dl), mean (±SE)* | 3.0 (0.1) | 2.6 (0.1) | 0.29 (0.13-0.65) | 0.28 (0.12-0.64) | 0.39 (0.14-1.05) | 0.06 |
| Glucose *(per 10 mg/dL increase), mean (±SE)* | 129.6 (5.1) | 134.2 (14.3) | 1.00 (0.90-1.11) | 1.00 (1.00-1.11) | 1.00 (0.90-1.11) | 0.79 |
| BMI *(kg/m²), mean (±SE)* | 25.2 (0.4) | 23.5 (0.9) | 0.92 (0.83-1.02) | 0.91 (0.81-1.03) | 0.94 (0.81-1.08) | 0.37 |
| ACB index*, mean (±SE)* | 20.9 (1.5) | 25.0 (2.8) | 1.00 (0.99-1.02) | 1.01 (0.99-1.02) | 1.01 (0.99-1.03) | 0.30 |
| Gender, *n (%)* |  |  |  |  |  |  |
| Female (Ref.) | 80 (48.5%) | 12 (41.4%) | 1.00 | 1.00 | 1.00 | 0.16 |
| Male | 85 (51.5%) | 17 (58.6%) | 1.33 (0.60-2.97) | 1.54 (0.67-3.54) | 2.13 (0.75-6.09) |  |
| Admission Year, *n (%)* |  |  |  |  |  |  |
| 2010-2016 (Ref.) | 46 (27.9%) | 10 (34.5%) | 1.00 | 1.00 | 1.00 | 0.07 |
| 2017-2023 | 119 (72.1%) | 19 (65.5%) | 0.73 (0.32-1.70) | 0.53 (0.21-1.31) | 0.36 (0.12-1.07) |  |
| LCA class, *n (%)* |  |  |  |  |  |  |
| No/Low comorbidity (Ref.) | 98 (59.4%) | 10 (34.5%) | 1.00 | 1.00 | 1.00 | 0.03* |
| Oncologic–liver disease | 19 (11.5%) | 4 (13.8%) | 2.06 (0.59-7.27) | 2.23 (0.60-8.24) | 2.78 (0.64-12.05) |  |
| Chronic multimorbidity with respiratory involvement | 48 (29.1%) | 15 (51.7%) | 3.06 (1.28-7.32) | 3.45 (1.38-8.60) | 4.34 (1.45-12.96) |  |

All models were developed using Multiple Imputation to handle missing data for Barthel Index, Albumin, Glucose and BMI.

Continuous variables are presented as mean (standard error) and categorical variables are expressed as frequencies (percentage). Odds ratios refer to a one-unit increase unless otherwise specified; for glucose, odds ratios refer to a 10 mg/dL increase; for the Barthel Index to a 10-point increase.

Global p-value of the multivariate model (chi-square test): 0.011.

SE Standard Error, OR Odds Ratio, CI Confidence Interval, LCA Latent Class Analysis, BMI Body Mass Index, ACB Anticholinergic Cognitive Burden, Ref. Reference Category.

^a^ p-value refers to the multivariate analysis: * p < 0.05, ** p ≤ 0.01, *** p ≤ 0.001.

**Supplementary Table S10** Association of patient characteristics with in-hospital mortality in sepsis diagnosed during hospitalization: logistic regression model (N = 252).

|  | **Discharged**  **(n = 161)** | **Dead**  **(n = 91)** | **Univariate**  **OR (95% CI)** | **Age and sex adjusted**  **OR (95% CI)** | **Multivariate**  **OR (95% CI)** | **p-value ^a^** |
| --- | --- | --- | --- | --- | --- | --- |
| Age *(years), mean (±SE)* | 79.8 (0.4) | 83.3 (0.7) | 1.06 (1.02-1.10) | 1.06 (1.02-1.09) | 1.05 (1.01-1.09) | 0.01** |
| Barthel index *(per 10-point increase),* *mean (±SE)* | 67.8 (2.8) | 53.8 (4.2) | **0.90 (0.82–1.00)** | **0.90 (0.82–1.00)** | 0.90 (0.90-1.00) | 0.20 |
| Albumin *(g/dl), mean (±SE)* | 3.1 (0.1) | 2.9 (0.1) | 0.52 (0.31-0.90) | 0.55 (0.31-0.95) | 0.60 (0.33-1.11) | 0.10 |
| Glucose *(per 10 mg/dL increase), mean (±SE)* | 132.6 (6.6) | 131.2 (7.8) | 1.00 (1.00-1.00) | 1.00 (1.00-1.00) | 1.00 (1.00-1.00) | 0.92 |
| BMI *(kg/m²), mean (±SE)* | 25.0 (0.4) | 24.7 (0.5) | 0.99 (0.93-1.05) | 1.00 (0.94-1.06) | 1.01 (0.95-1.07) | 0.82 |
| ACB index*, mean (±SE)* | 20.5 (1.5) | 28.0 (2.8) | **1.01 (1.00–1.02)** | **1.01 (1.00–1.02)** | **1.01 (1.00–1.02)** | 0.12 |
| Gender, *n (%)* |  |  |  |  |  |  |
| Female (Ref.) | 71.0 (44.1%) | 47.0 (51.6%) | 1.00 | 1.00 | 1.00 | 0.65 |
| Male | 90.0 (55.9%) | 44 (48.4%) | 0.74 (0.44-1.24) | 0.79 (0.47-1.34) | 0.88 (0.51-1.53) |  |
| Admission Year, *n (%)* |  |  |  |  |  |  |
| 2010-2016 (Ref.) | 57.0 (35.4%) | 35.0 (38.5%) | 1.00 | 1.00 | 1.00 | 0.41 |
| 2017-2023 | 104.0 (64.6%) | 56.0 (61.5%) | 0.88 (0.52-1.49) | 0.88 (0.51-1.51) | 0.78 (0.44-1.41) |  |
| LCA class, *n (%)* |  |  |  |  |  |  |
| No/Low comorbidity (Ref.) | 91.0 (56.5%) | 42.0 (46.2%) | 1.00 | 1.00 | 1.00 | 0.47 |
| Oncologic–liver disease | 26.0 (16.1%) | 17.0 (18.7%) | 1.42 (0.69-2.89) | 1.66 (0.79-3.47) | 1.61 (0.75-3.48) |  |
| Chronic multimorbidity with respiratory involvement | 44.0 (27.3%) | 32.0 (35.2%) | 1.58 (0.88-2.83) | 1.31 (0.71-2.40) | 1.09 (0.57-2.11) |  |

All models were developed using Multiple Imputation to handle missing data for Barthel Index, Albumin, Glucose and BMI.

Continuous variables are presented as mean (standard error) and categorical variables are expressed as frequencies (percentage). Odds ratios refer to a one-unit increase unless otherwise specified; for glucose, odds ratios refer to a 10 mg/dL increase; for the Barthel Index to a 10-point increase.

Global p-value of the multivariate model (chi-square test): 0.0289.

SE Standard Error, OR Odds Ratio, CI Confidence Interval, LCA Latent Class Analysis, BMI Body Mass Index, ACB Anticholinergic Cognitive Burden, Ref. Reference Category.

^a^ p-value refers to the multivariate analysis: * p < 0.05, ** p ≤ 0.01, *** p ≤ 0.001.

**Supplementary Table S11** Association of patient characteristics with length of stay (> 14 days) in sepsis diagnosed at admission: logistic regression model (N= 165)

| **Variable** | **LOS ≤ 14**  **(n = 117)** | **LOS > 14**  **(n = 48)** | **Univariate**  **OR (95% CI)** | **Age and sex adjusted**  **OR (95% CI)** | **Multivariate**  **OR (95% CI)** | **p-value ^a^** |
| --- | --- | --- | --- | --- | --- | --- |
| Age *(years), mean (±SE)* | 80.9 (0.7) | 79.4 (1.1) | 0.97 (0.93 -1.02) | 0.97 (0.93-1.02) | 0.97 (0.92-1.02) | 0.28 |
| Barthel index *(per 10-point increase),* *mean (±SE)* | 64.1 (3.3) | 66.1 (5.1) | 1.00 (0.99-1.01) | 1.00 (0.99-1.01) | 1.00 (0.99-1.01) | 0.94 |
| Albumin *(g/dl), mean (±SE)* | 3.0 (0.1) | 2.9 (0.1) | 0.6 (0.31-1.28) | 0.64 (0.32-1.30) | 0.79 (0.36-1.70) | 0.54 |
| Glucose *(per 10 mg/dL increase), mean (±SE)* | 132.6 (6.1) | 122.2 (7.6) | 1.00 (0.99-1.00) | 1.00 (0.99-1.00) | 1.00 (0.99-1.00) | 0.34 |
| BMI *(kg/m²), mean (±SE)* | 25.6 (0.5) | 24.6 (0.7) | 0.96 (0.89-1.04) | 0.96 (0.89-1.04) | 0.98 (0.89-1.06) | 0.57 |
| ACB index*, mean (±SE)* | 17.2 (2.4) | 29.7 (4.5) | 1.01 (1.00-1.03) | 1.01 (1.00-1.03) | 1.02(1.00-1.03) | 0.013* |
| Gender, *n (%)* |  |  |  |  |  |  |
| Female (Ref.) | 57 (48.7) | 23 (47.9) | Ref. | Ref. | Ref. | 0.59 |
| Male | 60 (51.3) | 25 (52.1) | 1.03 (0.53-2.02) | 1.01 (0.51-1.98) | 1.23 (0.58-2.60) |  |
| Admission Year, *n (%)* |  |  |  |  |  |  |
| 2010-2016 (Ref.) | 31 (26.5) | 15 (31.2) | Ref. | Ref. | Ref. | 0.45 |
| 2017-2023 | 86 (73.5) | 33 (68.8) | 0.79 (0.38 – 1.65) | 0.84 (0.39-1.80) | 0.73 (0.32-1.65) |  |
| LCA class, *n (%)* |  |  |  |  |  |  |
| No/Low comorbidity (Ref.) | 69 (59.0) | 29 (60.4) | Ref. | Ref. | Ref. | 0.28 |
| Oncologic–liver disease | 11 (9.4) | 8 (16.7) | 1.73 (0.63-4.74) | 1.79 (0.65-4.95) | 1.85 (0.63-5.46) |  |
| Chronic multimorbidity with respiratory involvement | 37 (31.6) | 11 (22.9) | 0.71 (0.27-1.58) | 0.69 (0.30-1.55) | 0.68 (0.28-1.66) |  |

All models were developed using Multiple Imputation to handle missing data for Barthel Index, Albumin, Glucose and BMI.

Continuous variables are presented as mean (standard error) and categorical variables are expressed as frequencies (percentage). Odds ratios refer to a one-unit increase unless otherwise specified; for glucose, odds ratios refer to a 10 mg/dL increase; for the Barthel Index to a 10-point increase.

Global p-value of the multivariate model (chi-square test): 0.329
